# Claudin-4 knockout by TALEN-mediated gene targeting in MDCK cells: Claudin-4 is dispensable for the permeability properties of tight junctions in wild-type MDCK cells

**DOI:** 10.1371/journal.pone.0182521

**Published:** 2017-08-04

**Authors:** Shinsaku Tokuda, Toyohiro Hirai, Mikio Furuse

**Affiliations:** 1 Department of Respiratory Medicine, Graduate School of Medicine, Kyoto University, Kyoto, Japan; 2 Division of Cell Structure, National Institute for Physiological Sciences, Okazaki, Japan; 3 Department of Physiological Sciences, SOKENDAI (The Graduate University for Advanced Studies), Okazaki, Japan; University of Chicago Medical Center, UNITED STATES

## Abstract

Epithelia act as a barrier between the internal and external environments, and the movement of substances via the paracellular pathway is regulated by tight junctions (TJs). Claudins are major determinants of TJ permeability. Claudin-4 was the first claudin whose involvement in the TJ permeability in cultured cells was directly demonstrated, but the permeability properties of individual claudins including claudin-4 are still incompletely clarified. In this study, we established claudin-4 knockout cells using transcription activator-like effector nucleases (TALENs), a recently developed method for genome editing, and investigated the permeability property of claudin-4 in MDCK II cells. We found that claudin-4 knockout has no apparent effect on the localization of other claudins and electrophysiological properties in MDCK II cells. Therefore we further established claudin-2 and claudin-4 double knockout clones and investigated the effects on TJs. Claudin-4 knockout in addition to claudin-2 knockout slightly increased the localization of other claudins at TJs but showed no obvious effects on the electrophysiological properties in MDCK II cells. These results indicate that claudin-4 is dispensable for the barrier property of TJs in wild-type as well as claudin-2 knockout MDCK II cells. Our results suggest the need for further knockout analysis to reveal the permeability properties of individual claudins.

## Introduction

In multicellular organisms, epithelia separate internal and external environments. The movement of substances across the epithelia is properly regulated, which contributes to the maintenance of homeostasis in the body. There are two routes for transepithelial transport: transcellular and paracellular pathways. In the paracellular pathway, the transport is regulated by tight junctions (TJs). TJs are one mode of cell-cell junctions located at the most apical part of junctional complexes [1,2], and ion permeability and charge selectivity in the TJs vary among epithelia [3,4].

The major determinants of TJ permeability are claudins, a large family of integral membrane proteins [5]. There are 27 claudin members in mammals [6], and the expression patterns of claudins determine a variety of permeability in TJs [7,8]. However, the permeability of individual claudins is still incompletely clarified. The effects of individual claudins on TJ permeability have been investigated by the overexpression or knockdown of the claudins in cultured epithelial cells. Nevertheless, since most epithelia express multiple different claudins, the endogenous claudins, which already constitute TJs, may mask the effects of overexpression or knockdown of claudin(s) on TJ permeability. Moreover, the overexpression of exogenous claudin(s) may cause displacement of endogenous claudins from TJs, which have a potential to affect TJ permeability. These problems make it difficult to reveal the permeability properties of individual claudins [8].

The permeability property of claudin-2 has been most well studied among all claudins. Many studies by the overexpression of claudin-2 in cultured cells have revealed that claudin-2 forms highly conductive channels with cation selectivity in TJs [9–12]. In addition, we recently showed that claudin-2 knockout using transcription activator-like effector nucleases (TALENs) increased transepithelial electrical resistance (TER) by more than 50-fold and transformed the so-called ‘leaky’ epithelia into ‘tight’ epithelia in MDCK II cells, indicating that claudin-2 independently determines the ‘leaky’ property of TJs in MDCK II cells [13].

TALENs are artificial nucleases generated by fusing a *Fok*I DNA cleavage domain to transcription activator-like effectors (TALEs) which bind specific nucleotides [14]. Two TALENs recognize the left and right arms of the target site and form a functional *Fok*I dimer. The *Fok*I dimer induces DNA double-strand breaks at the target site, resulting in the mutation and functional knockout of the gene [15].

Claudin-4 was the first claudin whose involvement in the TJ permeability in cultured cells was directly demonstrated. The overexpression of claudin-4 has been shown to increase TER and decrease cation selectivity in MDCK II cells [11,16]. These studies suggested that claudin-4 is likely to act as a Na^+^ barrier in TJs. However, since claudin-2 was indicated to have strong effects on ion permeability and cation selectivity in MDCK II cells [13], the decrease in Na^+^ permeability by the overexpression of claudin-4 might be caused by the displacement of claudin-2 from TJs.

In this study, we established claudin-4 knockout clones in MDCK II cells and investigated the permeability property of claudin-4.

## Results

### Establishment of claudin-4 knockout clones in MDCK II cells

**Fig 1:**
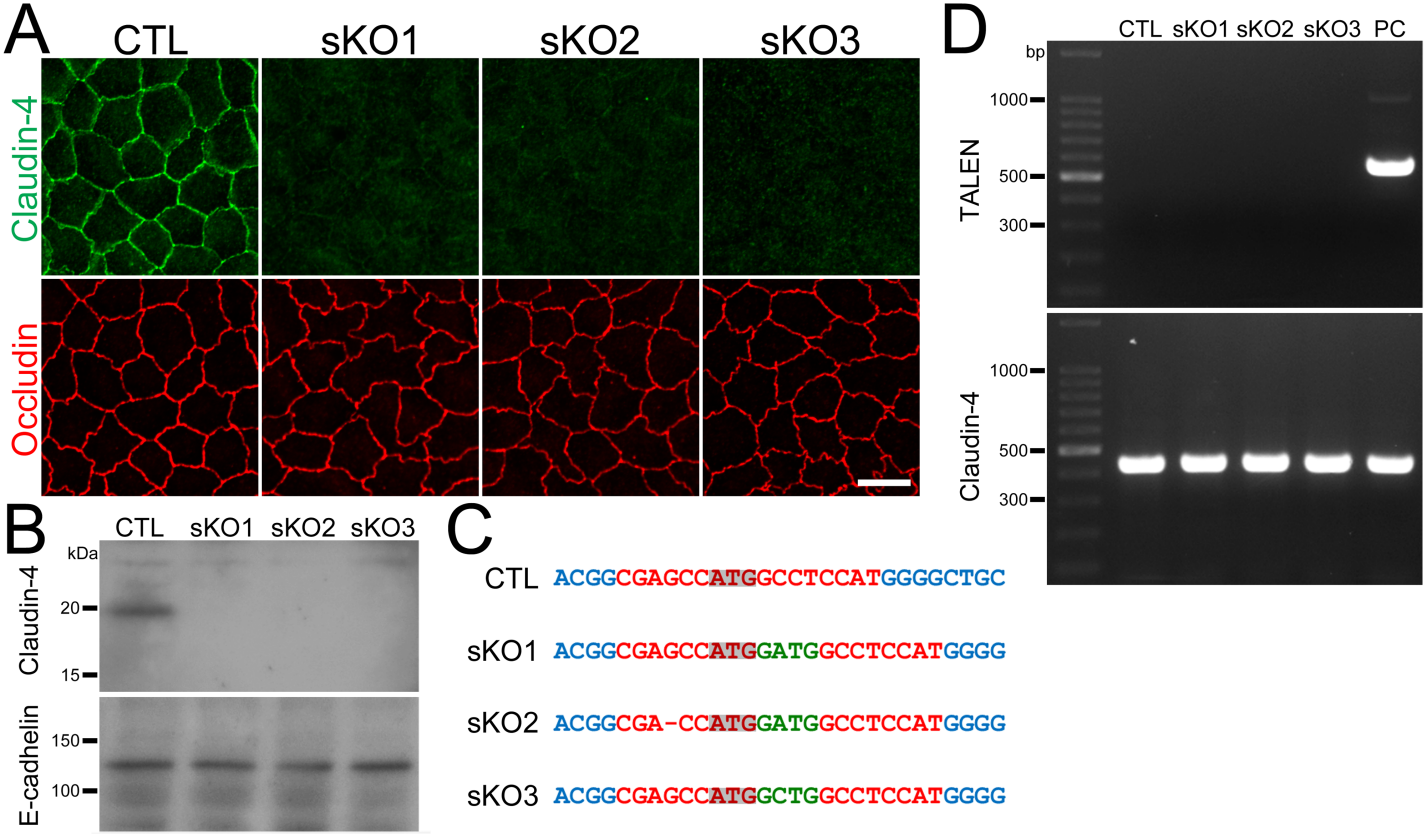
Establishment of claudin-4 knockout clones in MDCK II cells. (A) Immunofluorescence analysis of claudin-4 and occludin in wild-type cells (CTL) and claudin-4 knockout clones (sKO1–3) in MDCK II cells. Claudin-4 staining at cell-cell contacts was completely lost in claudin-4 knockout clones. Scale bar = 10 μm. (B) Immunoblots of claudin-4 and E-cadherin in wild-type cells and claudin-2 knockout clones. A claudin-4 band of ~20 kDa was absent in claudin-4 knockout clones. (C) DNA sequences of the TALEN targeting site in wild-type cells and claudin-4 knockout clones. Dash indicates loss of a nucleotide and green letters indicate additional nucleotides. Frame shifts were confirmed in all clones. (D) Genomic PCR analysis of wild-type cells and claudin-4 knockout clones using primers for TALEN and claudin-4 DNAs. A clone stably expressing TALEN was used as a positive control (PC). None of the PCR products for TALENs was detected in claudin-4 knockout clones.

To investigate the permeability property of claudin-4, we established claudin-4 knockout clones in MDCK II cells using TALENs constructed in a previously study [13]. The pair of TALEN DNA constructs targeting the left and right arms of the initiating codon in the canine claudin-4 were cloned into the vectors with neomycin- and puromycin-resistance genes and transfected into MDCK II cells. Then G418 and puromycin were transiently administered to select the transfected cells, and the remaining cells were screened by immunofluorescence microscopy as described previously [17]. Three independent clones were established, and immunofluorescence microscopy revealed complete disappearance of claudin-4 staining at cell-cell contacts in these clones (Fig 1A). Immunoblot analysis also showed complete loss of ~20 kDa bands representing claudin-4 in the clones (Fig 1B). To examine the mutations at the TALEN targeting site in claudin-4 genes, we performed DNA sequencing analysis for the PCR products from this site in the clones. Chromatograms of the sequences showed single peak arrays in all clones (S1 Fig), and sequence analysis revealed frame shifts in all claudin-4 genes in the clones (Fig 1C). These results indicate that the claudin-4 gene was successfully knocked out in these clones.

To confirm whether the TALEN constructs transfected into the clones were integrated into the chromosomes, we performed genomic PCR using the primers for the TALEN C-terminal region. A clone stably expressing the TALEN was used as a positive control [17]. The band of 558 bp representing TALENs was not detected in the clones (Fig 1D), suggesting that the TALEN constructs were not integrated into the chromosomes in these clones.

### Effects of claudin-4 knockout on the localization of other claudins

**Fig 2:**
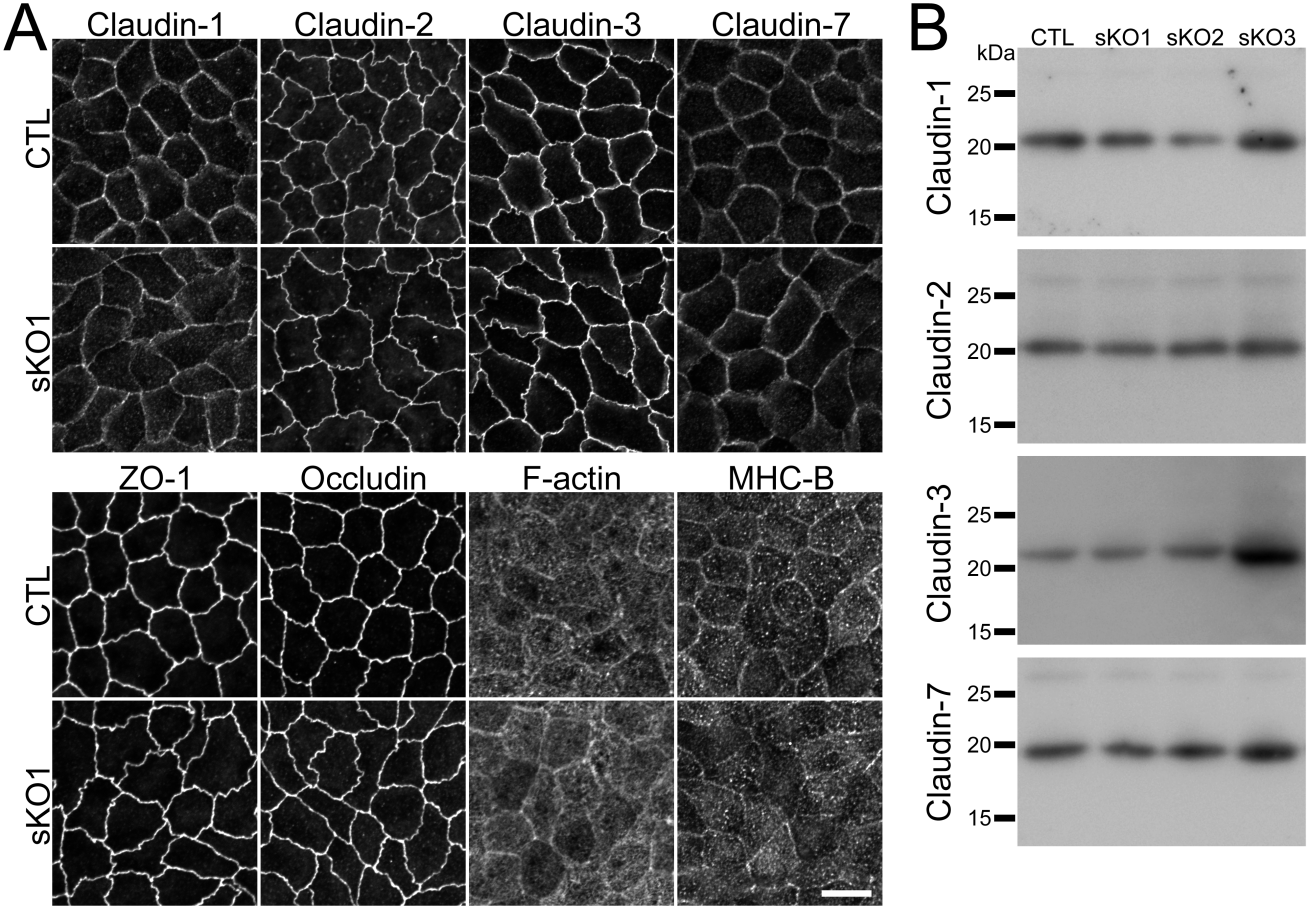
Effects of claudin-4 knockout on the localization of other TJ proteins and cytoskeleton. (A) Immunofluorescence analysis of claudin-1, -2, -3, -7, ZO-1, occludin, F-actin, and myosin heavy chain II-B (MHC-B) in wild-type cells (CTL) and claudin-4 knockout clone 1 (sKO1). The localization of these proteins was not apparently changed in the claudin-4 knockout clone. Scale bar = 10 μm. (B) Immunoblots of claudin-1, -2, -3, and -7 in wild-type cells and claudin-4 knockout clones.

MDCK II cells express claudin-1, -2, -3, and -7 as well as claudin-4 [17,18]. Since claudin-2 knockout has been reported to increase the localization of claudin-1, -3, -4 and -7 at TJs [13], we investigated the effects of claudin-4 knockout on the localization of other claudins. Immunofluorescence analysis showed that the localization of claudin-1, -2, -3 and -7 was similar in wild-type and claudin-4 knockout MDCK II cells (Fig 2A). The localization of ZO-1 (scaffold protein in TJs), occludin (integral membrane protein in TJs), F-actin and myosin was also examined, and the localization of these proteins was not apparently changed in claudin-4 knockout cells (Fig 2A).

**Fig 3:**
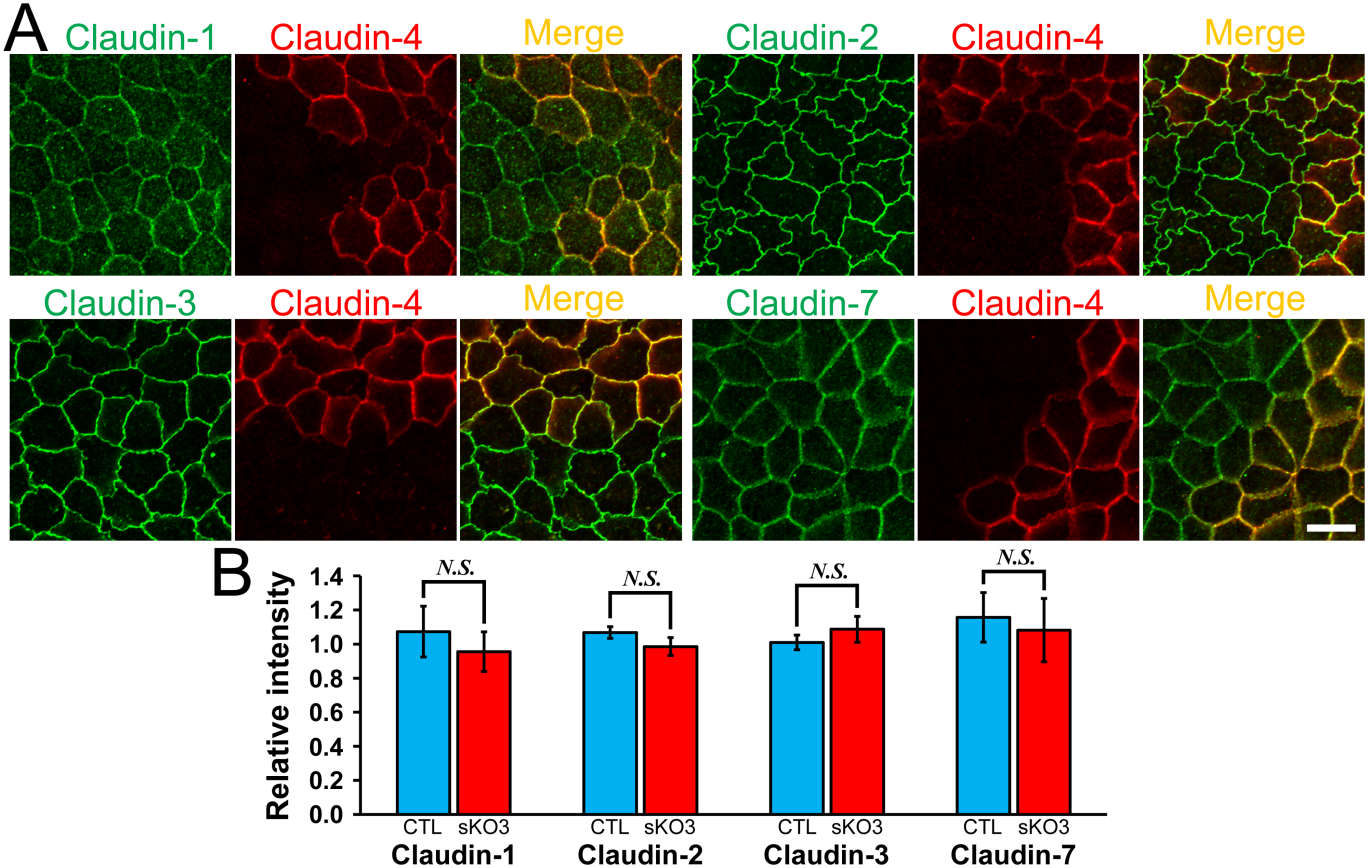
Effects of claudin-4 knockout on the localization of other claudins. (A) Immunofluorescence analysis of claudins in the co-culture of wild-type MDCK II cells and claudin-4 knockout clone 3 (sKO3). No apparent difference was observed between the localization of claudin-1, -2, -3 and -7 in the wild-type cells and claudin-4 knockout clone. Scale bar = 10 μm. (B) Quantification analysis of the signal intensity of claudins at TJs in wild-type cells and claudin-4 knockout clone. The signal intensity of claudins at TJs in wild-type cells and claudin-4 knockout clone 3 was measured as described in *Materials and Methods*, and the relative signal intensity of each claudin was calculated as the ratio of the signal intensity in wild-type cells (CTL) and claudin-4 knockout clone 3 (sKO3) to the signal intensity in wild-type cells. The signal intensity of claudins at TJs was not significantly different between wild-type cells and claudin-4 knockout clone. N = 4 for each experiment.

To compare the localization of claudins at TJs in wild-type and claudin-4 knockout cells in detail, we co-cultured the claudin-4 knockout clone with wild-type MDCK II cells. No apparent difference was observed between the localization of claudin-1, -2, -3 and -7 in the claudin-4 knockout clone and that in wild-type cells (Fig 3A). The quantification analysis showed that the signal intensity of claudins at TJs was not significantly changed by the claudin-4 knockout (Fig 3B). These results indicate that claudin-4 knockout has no apparent effect on the localization of other claudins.

We also checked the effects of claudin-4 knockout on the protein expression levels of claudin-1, -2, -3 and -7. The claudin-1 expression levels were slightly decreased in the sKO2 clone and increased in the sKO3 clone, and the claudin-3 expression level was increased in the sKO3 clone. The expression levels of claudin-1 and -3 in the other clones were similar to those in wild-type cells, and the expression levels of claudin-2 and -7 were not apparently changed in claudin-4 knockout clones (Fig 2B).

### Effects of claudin-4 knockout on the TJ barrier function

**Fig 4:**
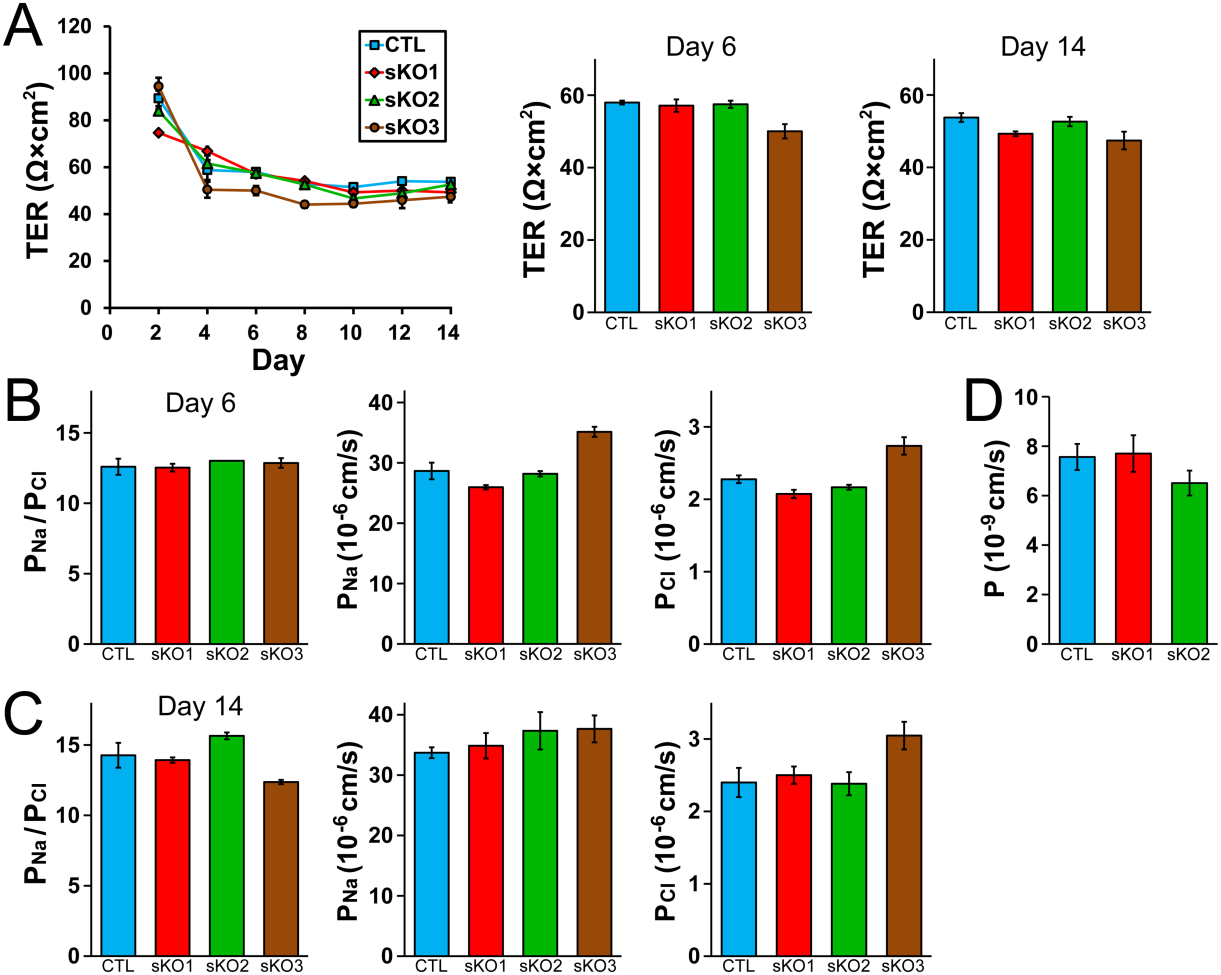
Effects of claudin-4 knockout on the barrier properties of TJs. (A) Time course of TER and TER values at 6 days and 14 days after the seeding on filter inserts in wild-type MDCK II cells (CTL) and claudin-4 knockout clones (sKO1–3). The time course of TER was similar in wild-type cells and claudin-4 knockout clones and the TER values at 6 days and 14 days after the seeding on filter inserts in claudin-4 knockout clones were comparable to those in wild-type cells. (B and C) Charge selectivity (the ratio of *P*_Na_ to *P*_Cl_: *P*_Na_/*P*_Cl_), *P*_Na_ and *P*_Cl_ at 6 days (B) and 14 days (C) after the seeding on filter inserts in wild-type cells and claudin-4 knockout clones. The values of *P*_Na_/*P*_Cl_, *P*_Na_ and *P*_Cl_ were similar in wild-type cells and claudin-4 knockout clones. (D) Flux of 4 kDa FITC-dextran in wild-type cells and claudin-4 knockout clones. Claudin-4 knockout had no significant effects on the flux of 4 kDa FITC-dextran. N = 3–4 for each experiment.

Next, we investigated the effects of claudin-4 knockout on the electrophysiological properties in MDCK II cells. The time course of TER was similar in wild-type cells and claudin-4 knockout clones (Fig 4A). The values of TER at 6 days and 14 days after the seeding on filter inserts in claudin-4 knockout clones were comparable to those in wild-type MDCK II cells (Fig 4A). We also measured charge selectivity and Na^+^ and Cl^-^ permeability across the epithelia (*P*_Na_/*P*_Cl_, *P*_Na_ and *P*_Cl_). Claudin-4 knockout clones as well as wild-type MDCK II cells showed high cation selectivity, and the values of *P*_Na_/*P*_Cl_, *P*_Na_ and *P*_Cl_ were similar in wild-type cells and claudin-4 knockout clones (Fig 4B and 4C). The permeability of 4 kDa FITC-dextran was also examined, and no significant difference was observed in wild-type cells and the claudin-4 knockout clones. These results indicate that claudin-4 knockout has no apparent effect on the TER, charge selectivity and the flux of 4 kDa dextran in MDCK II cells.

### Establishment of claudin-2 and claudin-4 double knockout clones in MDCK II cells

**Fig 5:**
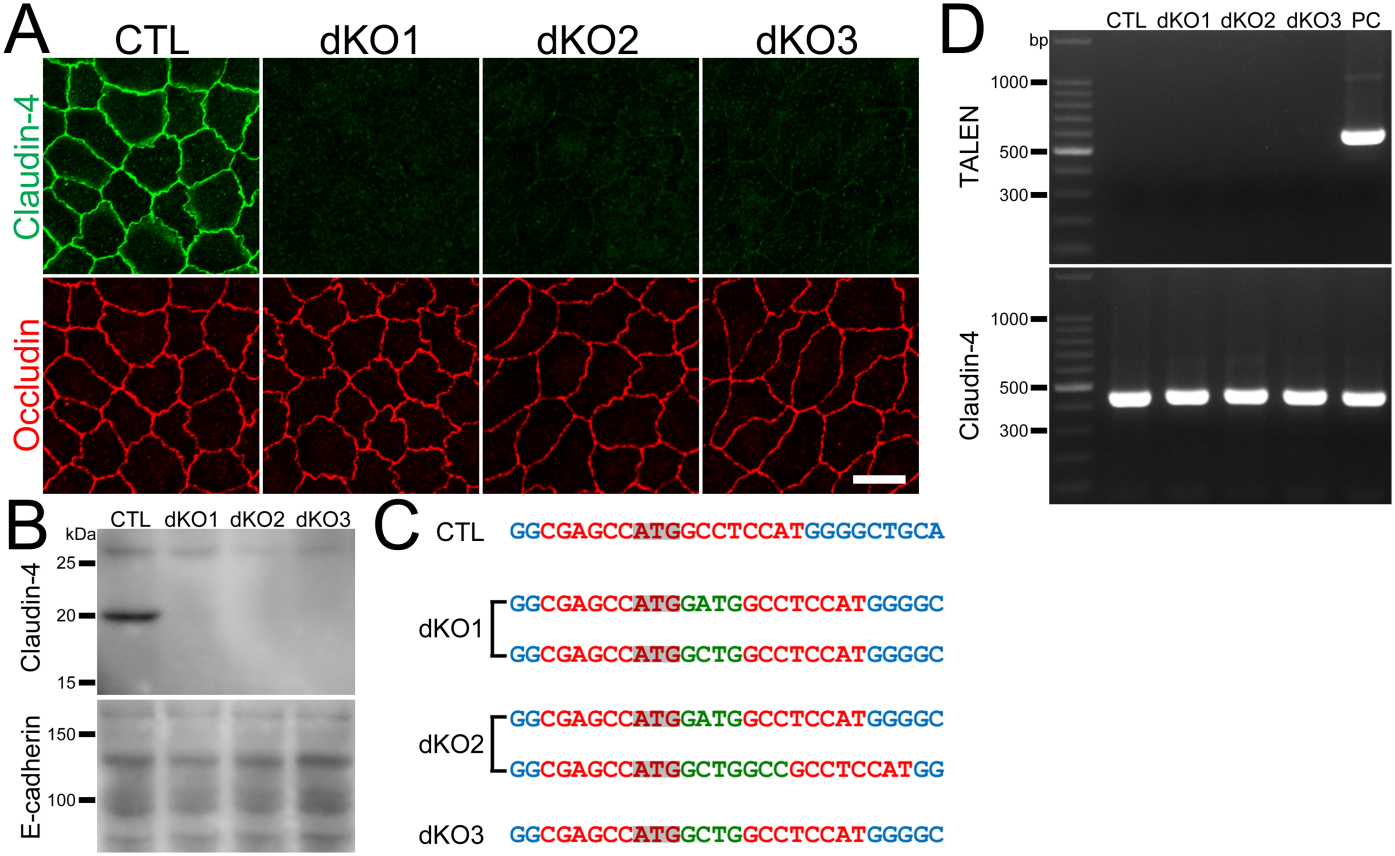
Establishment of claudin-2 and claudin-4 double knockout clones in MDCK II cells. (A) Immunofluorescence analysis of claudin-4 and occludin in claudin-2 knockout clone (CTL) and claudin-2 and claudin-4 double knockout clones (dKO1–3) in MDCK II cells. Claudin-4 staining at cell-cell contacts was completely lost in double knockout clones. Scale bar = 10 μm. (B) Immunoblots of claudin-4 and E-cadherin in claudin-2 knockout clone and double knockout clones. (C) DNA sequences of the TALEN targeting site in wild-type cells and double knockout clones. One type of mutation was found in the alleles of the dKO3 clone and two types in the alleles of the dKO1 and dKO2 clones. Green letters indicate additional nucleotides. Frame shifts were confirmed in all alleles. (D) Genomic PCR analysis of wild-type cells and double knockout clones using primers for TALEN and claudin-4 DNAs. None of the PCR products for TALENs was detected in double knockout clones.

The overexpression of claudin-4 has been reported to increase TER and decrease cation selectivity in MDCK II cells [11,16]. In contrast, claudin-4 knockout in this study had no apparent effect on TER and cation selectivity in MDCK II cells. Since claudin-2 is known to independently determine the highly conductive and cation selective property of TJs in wild-type MDCK II cells [13], there is a possibility that the endogenous claudin-4 is little involved in the TJ property in wild-type MDCK II cells. To further elucidate the permeability property of claudin-4, we established claudin-2 and claudin-4 double knockout clones and investigated the effects of the knockout on TJs. To avoid selecting biased clones, we transfected the TALENs for claudin-4 knockout into two different claudin-2 knockout clones (knockout clones 1 and 2 in a previous study [13]) and established two clones from claudin-2 knockout clone 1 (dKO1 and dKO2 clones) and one clone from claudin-2 knockout clone 2 (dKO3 clone). Immunofluorescence microscopy and immunoblot analysis revealed a complete loss of claudin-4 staining and bands in all clones (Fig 5A and 5B). Chromatograms of the sequences of the TALEN targeting site showed a single peak array in the dKO3 clone and mixed peak arrays in the dKO1 and dKO2 clones (S2 Fig). One peak was mixed in the chromatogram of the dKO1 clone. In contrast, the peaks after the initiating codon was mixed in the chromatogram of the dKO2 clone. Then we cloned the PCR products from the TALEN targeting site in the dKO2 clone into a plasmid vector for DNA sequencing analysis. Two patterns were observed in the chromatograms of the sequences, and the comparison of peak arrays revealed that the mixed peak array was composed of these two chromatograms (S2 Fig). Sequence analysis showed frame shifts in all claudin-4 alleles in the clones (Fig 5C). These results indicate successful claudin-4 gene knockout in these clones. We also confirmed whether the TALEN constructs transfected into the clones were integrated into the chromosomes, and the band representing TALENs was not detected in the clones (Fig 5D).

### Effects of claudin-2 and claudin-4 double knockout on the localization of other claudins

**Fig 6:**
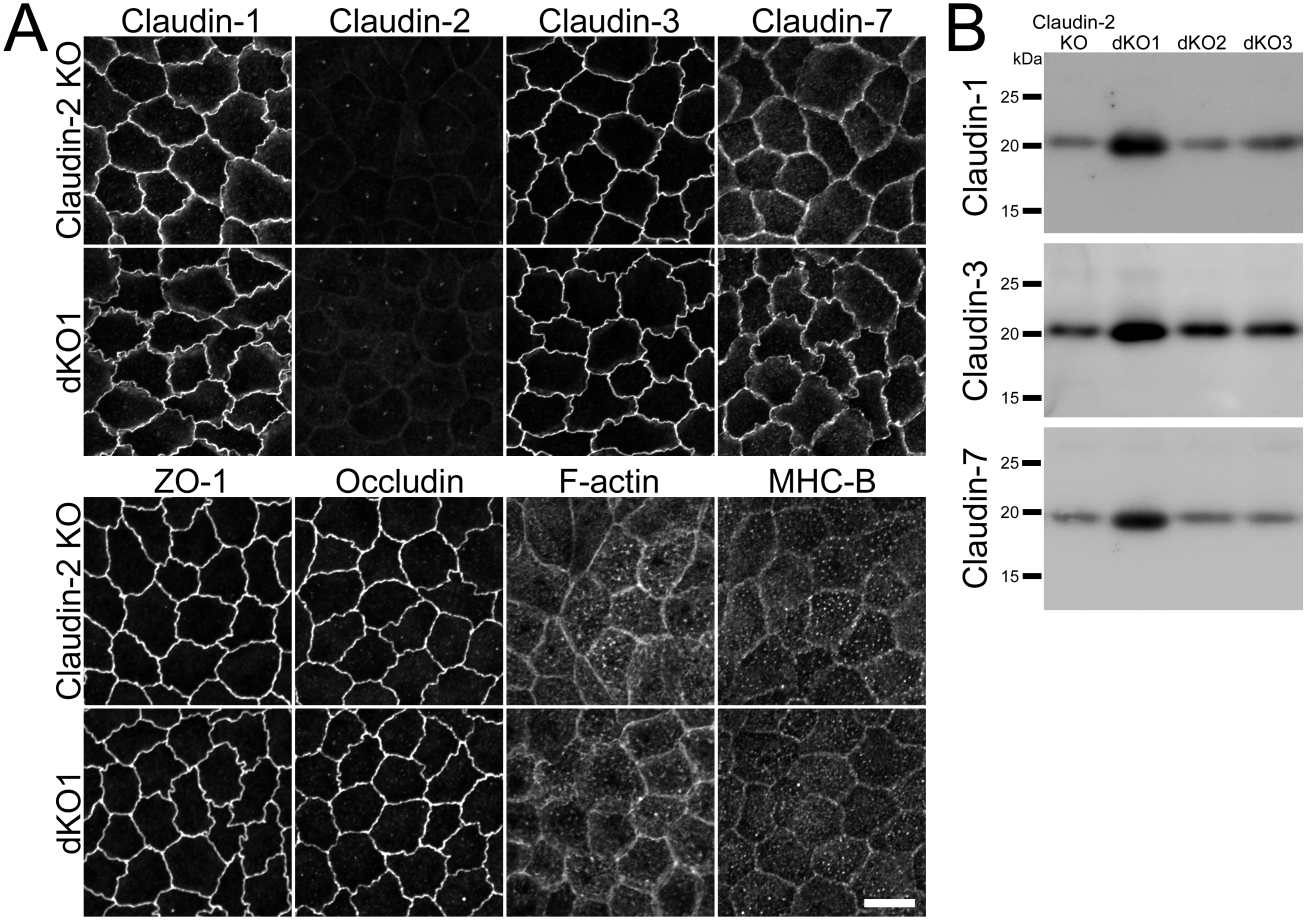
Effects of claudin-2 and claudin-4 double knockout on the localization of other TJ proteins and cytoskeleton. (A) Immunofluorescence analysis of claudin-1, -2, -3, -7, ZO-1, occludin, F-actin, and myosin heavy chain II-B (MHC-B) in claudin-2 knockout clone (claudin-2 KO) and claudin-2 and claudin-4 double knockout clone 1 (dKO1). The localization of claudin-7 in cell-cell contacts at the TJ level showed clearer sharp lines in the double knockout clone than the claudin-2 knockout clone. Scale bar = 10 μm. (B) Immunoblots of claudin-1, -3, and -7 in claudin-2 knockout clone and double knockout clones. The expression levels of claudin-1, -3 and -7 were increased in the dKO1 clone whereas those in the dKO2 and dKO3 clones were not changed compared with the claudin-2 knockout clone.

**Fig 7:**
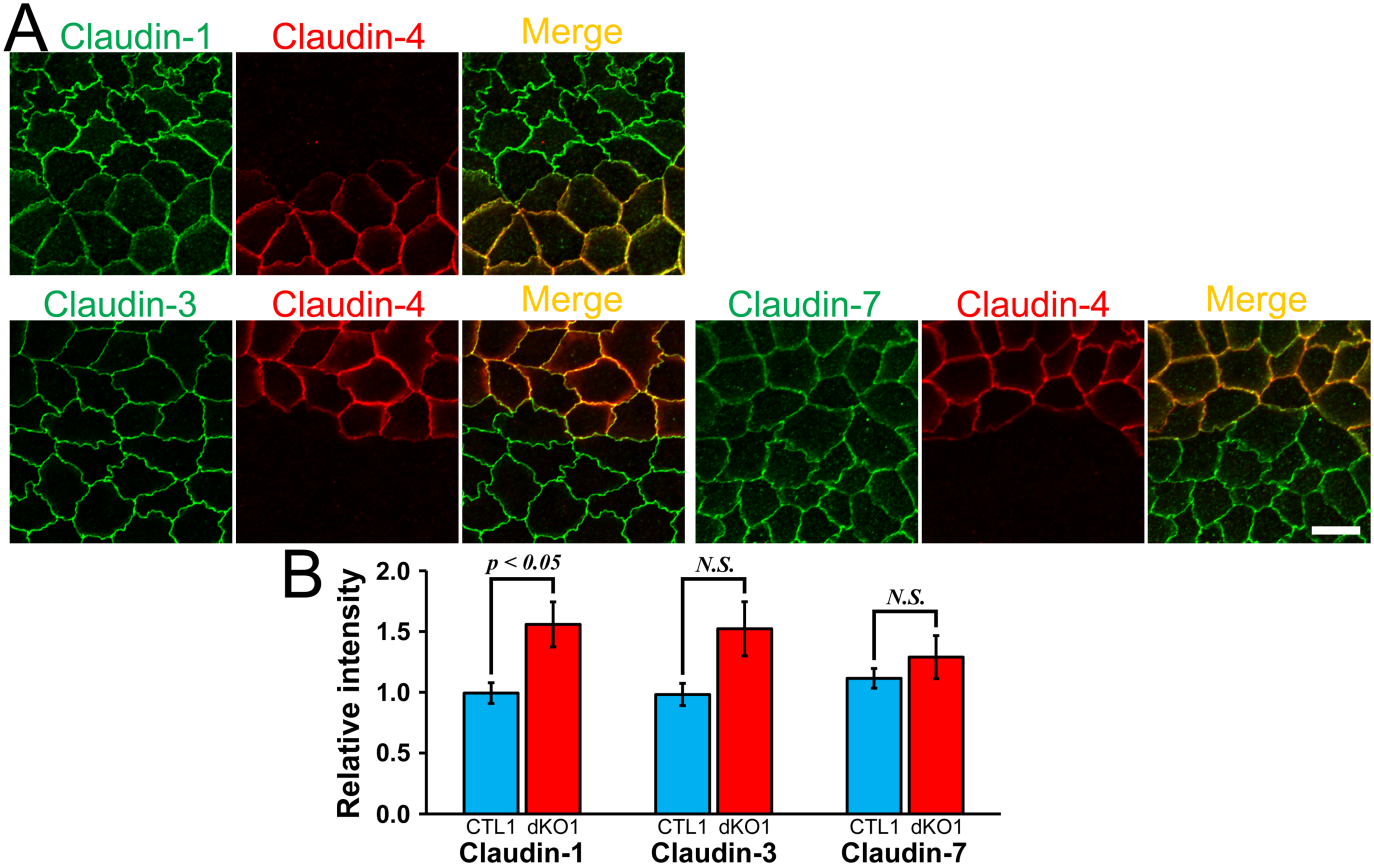
Effects of claudin-2 and claudin-4 double knockout on the localization of other claudins. (A) Immunofluorescence analysis of claudins in the co-culture of the claudin-2 knockout clone 1 [13] and the dKO1 clone (claudin-2 and claudin-4 double knockout clone). Scale bar = 10 μm. (B) Quantification analysis of the signal intensity of claudins at TJs in the claudin-2 knockout clone 1 (CTL1) and the dKO1 clone. N = 4 for each experiment.

Next, we investigated the effects of claudin-4 knockout, in addition to claudin-2 knockout, on the localization of other claudins. The localization of claudin-7 in cell-cell contacts at the TJ level showed clearer sharp lines in the claudin-2 and claudin-4 double knockout clone than in the claudin-2 knockout clone. The localization of other TJ proteins, F-actin and myosin was similar in the double knockout and claudin-2 knockout clones (Fig 6A). To confirm the effects of claudin-4 knockout in addition to claudin-2 knockout on the localization of other claudins in detail, the claudin-2 and claudin-4 double knockout clones were co-cultured with the claudin-2 knockout clones and observed by immunofluorescence microscopy (Fig 7A and S3A Fig). The signals of claudin-1, -3, and -7 in cell-cell contacts at the TJ level were slightly stronger in the double knockout clones than the claudin-2 knockout clones. The quantification analysis showed that the signal intensity of claudin-1, -3, and -7 at TJs was slightly higher in the double knockout clones than the claudin-2 knockout clones, although the signal intensity of claudin-3 and -7 at TJs in the dKO1 clone did not reach a level of significant difference compared with the claudin-2 knockout clone 1 (Fig 7B and S3B Fig). These results indicate that claudin-4 knockout in addition to claudin-2 knockout increases the localization of other claudins at TJs.

We also examined the effects of claudin-4 knockout in addition to claudin-2 knockout on the protein expression levels of other claudins. The expression levels of claudin-1, -3 and -7 were increased in the dKO1 clone, whereas those in the dKO2 and dKO3 clones were not changed compared with the claudin-2 knockout clone (Fig 6B).

### Effects of claudin-2 and claudin-4 double knockout on the TJ barrier function

**Fig 8:**
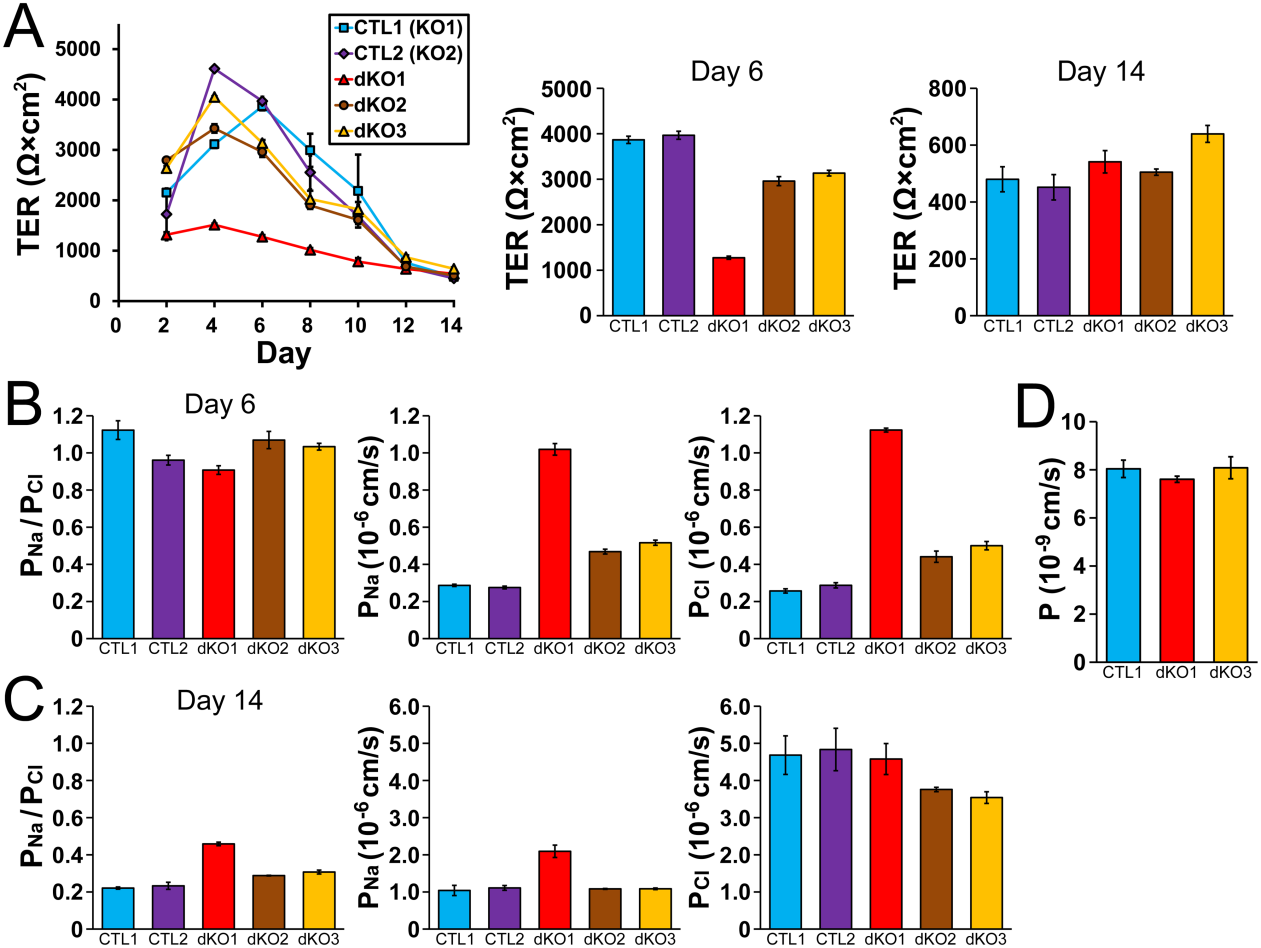
Effects of claudin-2 and claudin-4 double knockout on the barrier properties of TJs. (A) Time course of TER and TER values at 6 days and 14 days after the seeding on filter inserts in claudin-2 knockout clones (CTL1 and CTL2) and claudin-2 and claudin-4 double knockout clones (dKO1–3). (B and C) *P*_Na_/*P*_Cl_, *P*_Na_ and *P*_Cl_ at 6 days (B) and 14 days (C) after the seeding on filter inserts in claudin-2 knockout clones and claudin-2 and claudin-4 double knockout clones. (D) Flux of 4 kDa FITC-dextran in claudin-2 knockout clone and claudin-2 and claudin-4 double knockout clones. N = 3–4 for each experiment.

Next, we investigated the effects of claudin-4 knockout in addition to claudin-2 knockout on the electrophysiological properties in MDCK II cells. The TER of the claudin-2 knockout clones showed a peak at 4–6 days after the seeding on filter inserts, and the TER values at 6 days after the seeding were approximately 4000 Ω·cm^2^ (3866 ± 80 Ω·cm^2^ and 3967 ± 88 Ω·cm^2^; Fig 8A). The dKO2 and dKO3 clones showed similar time course of TER and the TER values at 6 days after the seeding were approximately 3000 Ω·cm^2^ (2959 ± 99 Ω·cm^2^ and 3134 ± 62 Ω·cm^2^), which were slightly lower but comparable to the TER values in claudin-2 knockout clones in a previous study (3000–4000 Ω·cm^2^ [13]). In contrast, the TER of the dKO1 clone was not sufficiently increased during the culture on filter inserts and the value at 6 days after the seeding was 1275 ± 34 Ω·cm^2^. The TER of the claudin-2 knockout and double knockout clones was then gradually decreased after a peak at 4–6 days after the seeding on filter inserts, and the TER at 14 days after the seeding showed similar values in all clones (claudin-2 knockout clone 1, 480 ± 44 Ω·cm^2^; claudin-2 knockout clone 2, 452 ± 45 Ω·cm^2^; dKO1 clone, 541 ± 39 Ω·cm^2^; dKO2 clone, 505 ± 11 Ω·cm^2^; dKO3 clone, 640 ± 30 Ω·cm^2^). The cation selectivity was markedly decreased at 6 days after the seeding in the claudin-2 knockout clones compared with wild-type cells, which is similar to the findings of a previous study [13]. Furthermore, the values of *P*_Na_/*P*_Cl_ at 14 days after the seeding showed further decreases in the claudin-2 knockout clones (claudin-2 knockout clone 1, 0.22 ± 0.01; claudin-2 knockout clone 2, 0.23 ± 0.02; Fig 8B and 8C). The cation selectivity in the claudin-2 and claudin-4 double knockout clones at 6 days after the seeding were also markedly decreased, and the values of *P*_Na_/*P*_Cl_ at 14 days after the seeding also showed further decreases, although the values were slightly higher than the claudin-2 knockout clones (dKO1 clone, 0.46 ± 0.01; dKO2 clone, 0.29 ± 0.00; dKO3 clone, 0.31 ± 0.01). To confirm whether the low values of TER at 2–10 days after the seeding on filter inserts in dKO1 clone is due to the lack of claudin-4, we performed rescue experiments. We transfected claudin-4 cDNA tagged with FLAG at N-terminus to the dKO1 clone and established stably expressing clones, although the expression levels of claudin-4 in these clones were relatively low for unknown reasons. The expression of claudin-4 did not restore the decrease of TER in dKO1 clone, suggesting that the decrease of TER in dKO1 clone is not due to the lack of claudin-4 (S4 Fig). These results indicate that TER shows a peak at 4–6 days after the seeding on filter inserts and then gradually decreases with an increase of anion selectivity in claudin-2 knockout MDCK II cells. Claudin-4 knockout, in addition to claudin-2 knockout, does not have obvious effects on these time course of TER and charge selectivity in claudin-2 knockout MDCK II cells, although there is a variation in the TER and charge selectivity among knockout clones.

There are two routes for the movement of substances across the epithelia: transcellular and paracellular pathways. In tight epithelia such as claudin-2 knockout MDCK II cells, it is necessary to take into consideration the contribution of the transcellular pathway in the measurements of charge selectivity and Na^+^ and Cl^-^ permeability across the epithelia [19,20]. To confirm the involvement of the transcellular pathway in the increase of anion selectivity at 14 days after the seeding on filter inserts in claudin-2 knockout cells, we examined the effects of 5-Nitro-2-(3-phenylpropylamino)benzoic acid (NPPB; chloride channel inhibitor) and bumetanide (Na^+^-K^+^-Cl^-^ cotransporter inhibitor) on *P*_Na_ and *P*_Cl_. The *P*_Na_ and *P*_Cl_ were slightly increased by the application of these inhibitors in the claudin-2 knockout clone while the *P*_Cl_ was slightly decreased by the application of these inhibitors in the claudin-2 and claudin-4 double knockout clone, but the anion selectivity remained to be observed after the application of the inhibitors in these clones (S5 Fig). These results suggest that the increase in anion selectivity at 14 days after the seeding on filter inserts in the claudin-2 knockout and the claudin-2 and claudin-4 knockout cells is likely to be at least partially due to the changes in the paracellular pathway. We further observed the localization of claudins at 14 days after the seeding on filter inserts in wild-type cells and knockout clones. The localization of claudins in wild-type cells and the claudin-4 knockout clone at 14 days after the seeding showed a similar appearance of those at 6 days after the seeding (S6 Fig). In contrast, local strong signals of claudin-1 and -7 were observed in some regions in cell-cell contacts in the claudin-2 knockout clone and the claudin-2 and claudin-4 double knockout clone at 14 days after the seeding on filter inserts. We also examined the permeability of 4 kDa FITC-dextran, and no significant difference was observed between the claudin-2 knockout clone and the claudin-2 and claudin-4 double knockout clones (Fig 8D).

**Fig 9:**
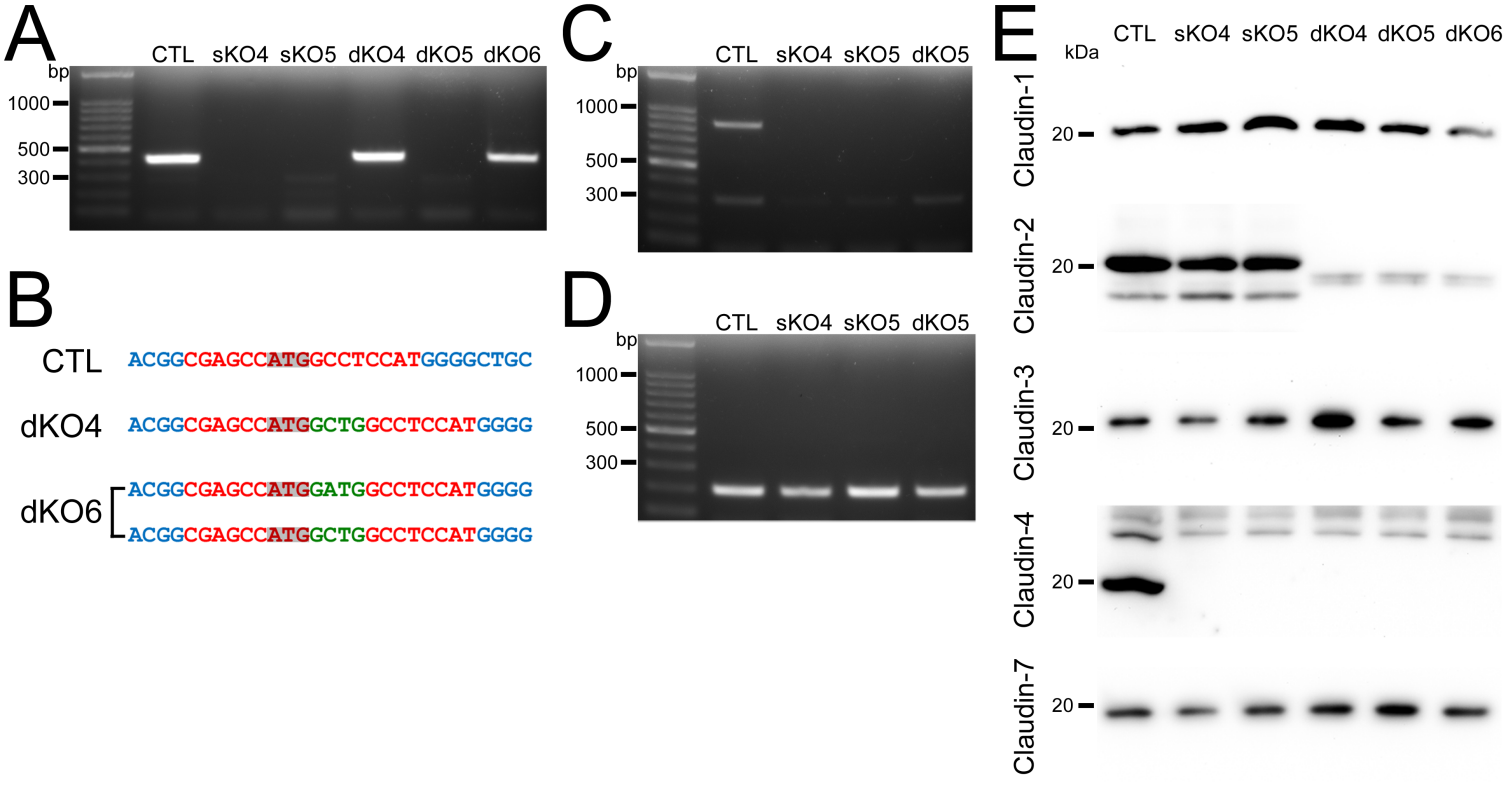
Establishment of claudin-2 and claudin-4 double knockout clones in MDCK II cells. (A) Genomic PCR analysis of wild-type cells, claudin-4 knockout clones (sKO4, sKO5) and claudin-2 and claudin-4 double knockout clones (dKO4–6) using primers for the TALEN targeting site in claudin-4 genes. (B) DNA sequences of the TALEN targeting site in wild-type cells and dKO4 and dKO6 clones. Green letters indicate additional nucleotides. (C) Genomic PCR analysis of wild-type cells and sKO4, sKO5 and dKO5 clones using primers for the region of sequences containing 400 bases before the TALEN targeting site. (D) Genomic PCR analysis of wild-type cells and sKO4, sKO5 and dKO5 clones using primers for the region within the claudin-4 gene. (E) Immunoblots of claudin-1, -2, -3, -4 and -7 in claudin-4 knockout clones.

The protein expression levels of claudin-1, -3, -7 were changed in some claudin-4 knockout clones (Figs 2B and 6B). To investigate whether these changes were due to the knockout of claudin-4 or not, we additionally established claudin-4 knockout clones and examined the protein expression levels of other claudins in these clones. We transfected the TALENs for claudin-4 knockout into wild-type MDCK II cells and claudin-2 knockout clone 2, screened the cells by immunofluorescence microscopy, and established two clones from wild-type cells (sKO4 and sKO5 clones) and three clones from claudin-2 knockout clone 2 (dKO4, dKO5 and dKO6 clones). To examine the mutations at the TALEN targeting site in claudin-4 genes, we performed PCR for this region. However, the PCR products were not detected in sKO4, sKO5 and dKO5 clones (Fig 9A). The PCR products of dKO4 and dKO6 clones were subjected to DNA sequencing analysis, and chromatograms of the sequences of the TALEN targeting site showed a single peak array in the dKO4 clone and mixed peak arrays in the dKO6 clone (S7 Fig). One peak was mixed in the chromatogram of the dKO6 clone. Sequence analysis showed frame shifts in all alleles of dKO4 and dKO6 clones (Fig 9B). We performed PCR for the region of sequences containing 400 bases before the TALEN targeting site. However, the PCR product was not detected in sKO4, sKO5 and dKO5 clones (Fig 9C). In contrast, PCR products for the region within the claudin-4 gene were detected in these clones (Fig 9D). These results suggested that massive nucleotides at the region before the TALEN target site was lost in sKO4, sKO5 and dKO5 clones. In immunoblot analysis, claudin-4 bands were completely lost in all knockout clones including sKO4, sKO5 and dKO5 clones (Fig 9E). Next, we examined the protein expression levels of claudins other than claudin-4 in these clones. The expression levels of claudin-1 were increased in the sKO5 clone and decreased in the dKO6 clone, those of claudin-3 were increased in the dKO4 and dKO6 clones and decreased in the sKO4 clone, and those of claudin-7 were increased in the dKO5 clone and decreased in the sKO4 clone (Fig 9E). The claudin-2 expression levels were not apparently changed in the sKO4 and sKO5 clones. These results indicate that claudin-4 knockout sometimes affects the expression levels of other claudins, although the type of claudins and the direction of changes affected by the claudin-4 knockout vary among clones.

## Discussion

In this study, we established claudin-4 knockout clones in MDCK II cells and investigated the permeability property of claudin-4. Claudin-4 knockout showed no apparent effect on the TER, *P*_Na_ and *P*_Cl_ in wild-type MDCK II cells, suggesting that endogenous claudin-4 is little involved in the barrier property of TJs in wild-type MDCK II cells. The barrier properties of TJs in wild-type MDCK II cells is known to be independently determined by claudin-2 [13], and the results in claudin-4 knockout MDCK II cells in this study seem consistent with this report. In contrast, the overexpression of claudin-4 has also been reported to increase TER and decrease cation selectivity in MDCK II cells [11,16]. However, since claudin-4 overexpression induced more complex TJ strands with the increase in strand number [16], the increase in TER and the decrease in cation selectivity are likely to be caused by the excessive TJ strands by the exogenous claudin-4. In addition, the overexpression of claudin-4 might also cause displacement of the endogenous claudin-2 from TJ strands and result in the changes in TJ properties [8]. To further analyze the permeability property of claudin-4, we established claudin-2 and claudin-4 double knockout clones and investigated the effects of knockout on TJs. However, claudin-4 knockout, in addition to claudin-2 knockout, also had no obvious effects on TER and charge selectivity. The claudin-2 and claudin-4 double knockout cells showed high TER values at 4–6 days after the seeding on filter inserts, and then the TER gradually decreased with an increase of anion selectivity. These results suggest that claudin-4 is dispensable for the barrier properties of TJs in wild-type and claudin-2 knockout MDCK II cells. Since claudin-2 and claudin-4 double knockout MDCK II cells still have the expression of at least claudin-1, -3 and -7, the remaining claudins might mask the effects of claudin-4 knockout in this study. Further analysis of the electrophysiological properties in claudin knockout cells including triple and quadruple knockouts of claudins may aid our understanding of the permeability properties of claudin-4 and other claudins.

The claudin-2 knockout and the claudin-2 and claudin-4 double knockout MDCK II cells showed high TER values at 4–6 days after the seeding on filter inserts, suggesting that claudin-1, -3 and/or -7 form TJs with high TER, although MDCK II cells may also express other claudins. Since claudin-1 and -3 have been reported to increase TER when overexpressed in MDCK II cells [21,22], these claudins may be involved in high TER in the claudin-2 knockout and the claudin-2 and claudin-4 double knockout MDCK II cells.

Interestingly, the TER in the claudin-2 knockout and the claudin-2 and claudin-4 double knockout MDCK II cells gradually decreased after a peak at 4–6 days after the seeding on filter inserts with an increase in anion selectivity. In tight epithelia such as the claudin-2 knockout MDCK II cells, it is necessary to take into consideration the contribution of the transcellular pathway in the measurements of charge selectivity and Na^+^ and Cl^-^ permeability across the epithelia [19,20]. Therefore we examined the effects of the inhibitors for the transepithelial transport of chloride on the electrophysiological properties in the claudin-2 knockout and the claudin-2 and claudin-4 double knockout MDCK II cells at 14 days after the seeding on filter inserts, and the anion selectivity remained to be observed after the application of the inhibitors. These results suggest that the paracellular pathway is likely to be involved in the increase of anion selectivity in the claudin-2 knockout and the claudin-2 and claudin-4 double knockout MDCK II cells, and that claudin-1, -3 and/or -7 may form anion selective channels in the paracellular pathway. Since claudin-7 has shown the possibility to act as anion channels [18], claudin-7 may be involved in the increase of anion selectivity in the claudin-2 knockout and the claudin-2 and claudin-4 double knockout MDCK II cells. In immunofluorescence microscopy, local strong signals of claudin-7 were observed at some regions in cell-cell contacts in the claudin-2 knockout and the claudin-2 and claudin-4 double knockout MDCK II cells at 14 days after the seeding on filter inserts, but the meaning of the observation was unclear. Further analysis of the effects of claudin-1, -3 and -7 knockout is required to understand the electrophysiological properties of TJs in MDCK II cells.

Claudin-4 knockout showed no apparent effects on the localization of other claudins in wild-type MDCK II cells. Since the electrophysiological properties of TJs have been reported to be independently determined by claudin-2 in wild-type MDCK II cells [13], TJ strands in wild-type MDCK II cells are likely to be mainly composed of claudin-2, whereas claudin-4 is less localized at TJs. There have been only a few reports about the regulation of claudin localization at TJs [23–25], and the mechanisms which determine the order of the preference to localize at TJs among claudin types is still poorly understood. Further analysis of the specific regions in claudins that determine the preference to localize at TJs using claudin-2 knockout MDCK II cells may provide clues for the elucidation of these mechanisms.

Genomic PCR analysis showed that TALEN DNAs were not integrated into the chromosomes in the knockout clones established in this and previous studies [13,17]. The persistent expression of TALENs may potentially increase the frequency of off-target cleavage especially in the case of double- and triple-gene knockouts by TALENs as described in detail previously [13,26]. Therefore, the knockout clones established in this and previous studies are likely to be useful for further knockout analyses.

The protein expression levels of claudin-1, -3 and -7 were changed in some claudin-4 knockout clones, although the type of claudins and the direction of changes affected by the claudin-4 knockout varied among clones. We previously established three ZO-1 knockout clones and five claudin-2 knockout clones, and the expression levels of claudins were not changed in these clones except for the expression levels of claudin-2 in claudin-2 knockout clones [13,17]. Therefore, the changes of claudin expression levels in claudin-4 knockout clones in this study are thought to be caused by the knockout of claudin-4, although the changes do not show a fixed tendency. The overexpression of claudin-4 in wild-type MDCK II cells has been reported to have no effect on the expression levels of other claudins [16]. Thus, the changes in the expression level of claudin-4 is not likely to induce fixed changes in the expression levels of other claudins, and the abrupt disappearance of claudin-4 by the knockout of claudin-4 may affect the expression levels of other claudins. Further analysis of the effects of claudin-4 knockout on the transcription factors involved in the regulation of claudin expression might increase our understanding of this phenomenon.

In conclusion, we established claudin-4 knockout clones and claudin-2 and claudin-4 double knockout clones in MDCK II cells. We found that claudin-4 is dispensable for the barrier property of TJs in wild-type as well as claudin-2 knockout MDCK II cells. Further knockout analysis is required to reveal the permeability properties of individual claudins in future studies.

## Materials and methods

### Cells, antibodies and reagents

MDCK II cells were provided by Dr. Masayuki Murata [9]. Cells were grown in DMEM (high glucose) supplemented with 5% fetal bovine serum. Mouse anti-ZO-1 monoclonal antibody (mAb) (T8/754), rat anti-occludin mAb (MOC37), rabbit anti-claudin-2 polyclonal antibody (pAb), and rabbit anti-claudin-4 pAb were characterized as described previously [27–30]. Rabbit anti-claudin-1 pAb (51–9000), mouse anti-claudin-2 mAb (32–5600), rabbit anti-claudin-3 pAb (34–1700), mouse anti-claudin-4 mAb (32–9400), rabbit anti-claudin-7 pAb (34–9100), and alexa fluor 488 phalloidin (A12379) were purchased from Invitrogen. Rabbit anti-nonmuscle myosin heavy chain II-B (MHC-B) pAb (PRB-445P) was purchased from Covance. Mouse anti-E-cadherin mAb (ECCD-2; M108) was purchased from Clontech. Fluorescein isothiocyanate-dextran (FITC-dextran), bumetanide (B3023) and NPPB (N4779) were purchased from Sigma-Aldrich.

### Establishment of knockout clones

Claudin-4 knockout clones were established in a similar manner as described previously [13,17]. In brief, a pair of TALEN constructs for the claudin-4 knockout were cloned into a mammalian expression vector pCAGGS [31] with a neomycin resistance gene and puromycin resistance gene, and transfected into cells. Then G418 and puromycin were administered transiently, and remaining clones were isolated and screened for claudin-4 depletion by immunocytochemistry.

### cDNA cloning and plasmid construction

cDNA encoding dog claudin-4 was cloned into pCAGGS with N-terminal 1×FLAG (DYKDDDDK) tag and 2StrepII (WSHPQFEK) tags. To establish stably expressing clones, the vectors were transfected into cells and stable clones were selected in standard media supplemented with 500 μg/ml G418.

### PCR amplification of genomic DNA

Genomic DNA was isolated by the Hot-shot method [32] and subjected to PCR for the amplification of TALEN targeting site in the claudin-4 gene (Forward: 5′-CTGGCTGAAAGGAACTGGTC-3′; Reverse: 5′-TACACCTTGCACTGCATCTG-3′), the region of sequences containing 400 bases before the TALEN targeting site (Forward: 5′-GGCAGTTTCCCCTGAGAC-3′; Reverse: 5′-GTCCCGGATGATATTGTTGG-3′), the region within the claudin-4 gene (Forward: 5′-CTCATGGTCGTCAGCATCAT-3′; Reverse: 5′-GTCCCGGATGATATTGTTGG-3′), and TALEN C-terminal region (Forward: 5′-CTGCGGCACAAATTGAAATA-3′; Reverse: 5′- ATGAGCGGAAATTGATCTCG-3′). The PCR products of TALEN targeting site in the claudin-4 gene were directly subjected to sequencing analysis. For the dKO2 clone, PCR products were cloned into pCAGGS and subjected to sequencing analysis.

### DNA sequencing analysis

DNA sequencing was performed using the dideoxy chain termination method with BigDye Terminator version 3.1 Cycle Sequencing Kit (Applied Biosystems) and the results were analyzed by the Applied Biosystems 3130 Genetic Analyzers (Applied Biosystems). The chromatograms of the sequence results were analyzed using Peak Scanner Software 2 (Applied Biosystems).

### Immunocytochemistry

Immunocytochemistry was performed as described previously [13]. In brief, cells were cultured on 12-mm-diameter Transwell filter inserts with a 0.4-μm pore size (Corning, Corning, NY) for 6 d unless otherwise noted, and filter inserts were fixed in 1% paraformaldehyde or in 100% methanol. Then the filters were permeabilized in a solution of 0.2% (w/v) Triton X-100 (EMD Biosciences). This was followed by blocking with 2% bovine serum albumin and incubation with a primary Ab and then by a fluorescence-labeled secondary Ab. The samples were imaged on a Zeiss LSM700 confocal microscope using a 63× Plan Apo lens. Contrast adjustment was generated using Adobe Photoshop (ver. 7.0).

### Immunoblotting

The Laemmli SDS sample buffer was added to the filter inserts. Then the epithelial cells on filter inserts were scraped in the sample buffer, and the buffer was collected into the microtube and boiled for 5 min. The proteins were then separated using the one-dimensional SDS-PAGE and electrotransferred from the gels to PVDF membranes followed by the incubation with primary Abs. The bound Abs was detected via HRP-linked secondary Abs and visualized by enhanced chemiluminescence (ECL Prime Kit; GE Healthcare).

### Barrier assays: Electrophysiological measurements and tracer flux

Electrophysiological studies were performed as described previously [13,33]. In brief, cells were plated at a density of 2 × 10^5^ cells/cm^2^ on 12-mm-diameter Transwell filter inserts with a 0.4-μm pore size, and the culture medium was exchanged every two days. Electrical resistance across the cell monolayer was measured using Millicell-ERS epithelial volt-ohm meter (Millipore) every two days, and transepithelial electrical resistance (TER) was determined by the subtraction of the resistance of the blank filter. Dilution potentials and TER of cell monolayers were measured, and the *P*_Na_/*P*_Cl_ ratio was calculated using the Goldman–Hodgkin–Katz equation. The values of *P*_Na_ and *P*_Cl_ were then calculated from the TER and *P*_Na_/*P*_Cl_ using the Kimizuka–Koketsu equation [34]. For transcellular transport-inhibitor studies, the epithelia were incubated in the buffer solutions with 100μM NPPB and 100μM bumetanide in apical and basal sides for 10 min at 37°C. *P*_Na_ and *P*_Cl_ were measured before and 10 min after the administration of the inhibitors.

For measurements of tracer flux, cell monolayers cultured for 6 d were incubated in the buffer solutions with 0.2 mM FITC-dextran in the basal side for 1 h, and the solutions in the apical side were collected. Fluorescence of the solutions was measured using a fluorescence spectrophotometer (F-4500; Hitachi High-Tech) and the amounts of FITC–dextran were determined by extrapolation from a standard curve. The permeability of FITC-dextran was defined as (dQ/dt)/AC_0_ [13,35].

### Quantification of the localization of TJ proteins at the TJs

We quantified the signal intensity of TJ proteins at TJs using Image J 1.43u as previously described [13,36]. We performed triple staining for the target proteins with occludin (TJ marker protein) and claudin-4 (knockout protein), and single confocal images of triple-stained monolayers at the level of TJs were captured. Images were opened in Image J 1.43u, and immunofluorescence signals of occludin were traced with 0.5 μm-wide freehand lines to build the region of interest. We traced five sides of control cells and claudin-4 knockout cells in one image, and integrated density of pixel gray values of ROI was calculated (D_CTL_ and D_KO_). To estimate the localization of claudins at TJs in claudin-4 knockout cells, the relative intensity of each claudin was calculated as D_KO_/D_CTL_.

### Statistical analysis

Data are represented as means ± standard error of the mean. The statistical analysis was performed using the Student’s *t*-test for comparison of two means and Bonferroni correction for multiple comparison. P < 0.05 was considered statistically significant.

## Supporting information

S1 Fig Chromatograms of sequences around the TALEN targeting site in wild-type cells and claudin-4 knockout clones. PCR products of the TALEN targeting site from wild-type cells (CTL) and claudin-4 knockout clones (sKO1–3) were directly subjected to DNA sequencing analysis. Chromatograms of the sequences showed single peak arrays in the knockout clones.
(TIF)

S2 Fig Chromatograms of sequences around the TALEN targeting site in wild-type cells and claudin-2 and claudin-4 double knockout clones. PCR products of the TALEN targeting site from wild-type cells (CTL) and claudin-2 and claudin-4 double knockout clones (dKO1–3) were directly subjected to DNA sequencing analysis. Chromatograms of the sequences of the TALEN targeting site showed a single peak array in the dKO3 clone and mixed peak arrays in the dKO1 and dKO2 clones. PCR products from the dKO2 clone were cloned into a plasmid vector and subjected to sequencing analysis.
(TIF)

S3 Fig Effects of claudin-2 and claudin-4 double knockout on the localization of other claudins. (A) Immunofluorescence analysis of claudins in the co-culture of the claudin-2 knockout clone 2 [13] and the dKO3 clone (claudin-2 and claudin-4 double knockout clone). Scale bar = 10 μm. (B) Quantification analysis of the signal intensity of claudins at TJs in the claudin-2 knockout clone 2 (CTL2) and the dKO3 clone. N = 4 for each experiment.
(TIF)

S4 Fig Effects of claudin-4 re-expression on electrophysiological properties in the dKO1 clone. (A) Immunofluorescence analysis of claudin-4 and occludin in claudin-2 knockout clone 1 (CTL), dKO1 clone, and rescue clone. Claudin-4 cDNA was transfected into dKO1 clone, and the clone expressing N-terminally FLAG tagged claudin-4 was established. Scale bar = 10 μm. (B) Time course of TER in claudin-2 knockout clone 1, dKO1 clone, and rescue clone. (C and D) *P*_Na_/*P*_Cl_, *P*_Na_ and *P*_Cl_ at 6 days (C) and 14 days (D) after the seeding on filter inserts in claudin-2 knockout clone 1, dKO1 clone, and rescue clone. N = 3–4 for each experiment.
(TIF)

S5 Fig Effects of the inhibitors of transcellular transport on *P*_Na_ and *P*_Cl_ in claudin-2 knockout clone and claudin-2 and claudin-4 double knockout clone at 14 days after the seeding on filter inserts. Claudin-2 knockout clone and claudin-2 and claudin-4 double knockout clone were cultured for 14 days on filter inserts. *P*_Na_ and *P*_Cl_ were measured before (−) and 10 min after (+) the administration of 100μM NPPB and 100μM bumetanide in both the apical and basal sides.
(TIF)

S6 Fig Immunofluorescence analysis of claudins in wild-type cells and claudin knockout clones at 14 days after the seeding on filter inserts. Wild-type MDCK II cells, claudin-4 knockout clone, claudin-2 knockout clone, and claudin-2 and claudin-4 double knockout clone were cultured for 14 days on filter inserts and analyzed by immunofluorescence microscopy for claudins. Scale bar = 10 μm.
(TIF)

S7 Fig Chromatograms of sequences around the TALEN targeting site in wild-type cells and claudin-4 knockout clones. PCR products of the TALEN targeting site from wild-type cells (CTL) and claudin-4 knockout clones (dKO4, dKO6) were directly subjected to DNA sequencing analysis.
(TIF)
