# Supplementary figures and images for: Claudin-4 knockout by TALEN-mediated gene targeting in MDCK cells: Claudin-4 is dispensable for the permeability properties of tight junctions in wild-type MDCK cells
Source: PLoS One. 2017 Aug 4;12(8):e0182521. doi: 10.1371/journal.pone.0182521 (PMC5544209; doi:10.1371/journal.pone.0182521)

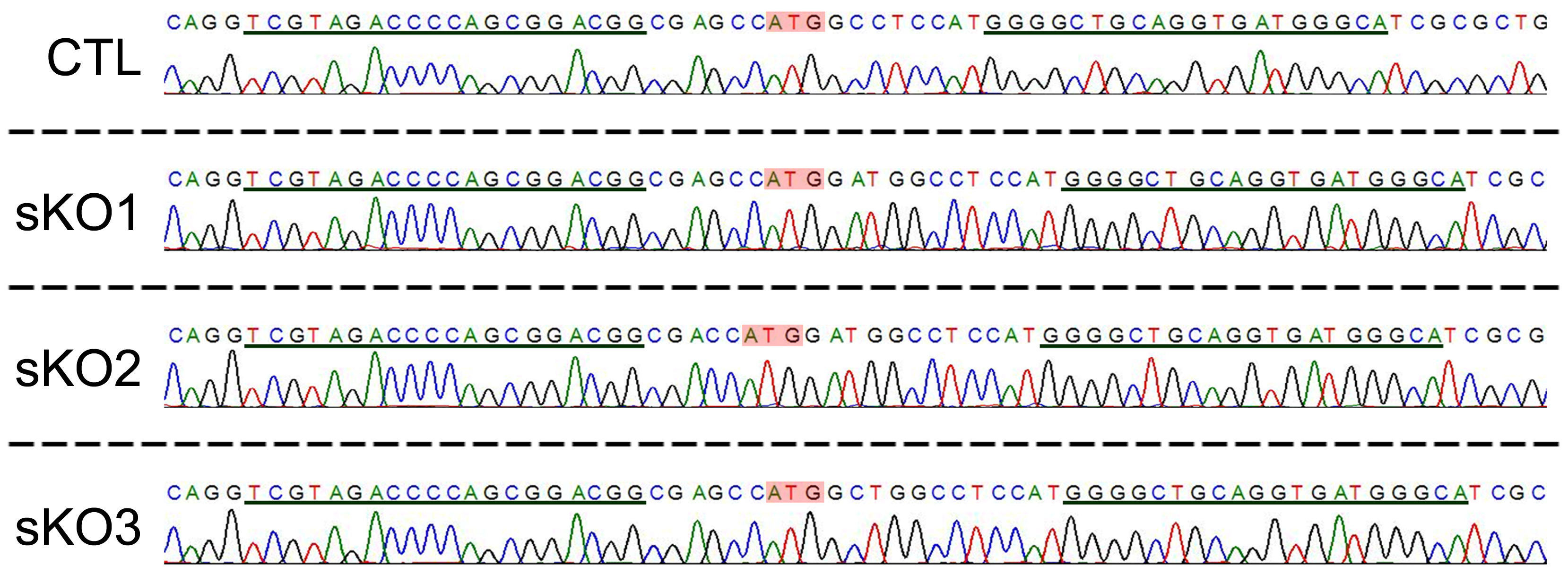

Supplement: S1 Fig — PCR products of the TALEN targeting site from wild-type cells (CTL) and claudin-4 knockout clones (sKO1–3) were directly subjected to DNA sequencing analysis. Chromatograms of the sequences showed single peak arrays in the knockout clones. (TIF) [file pone.0182521.s001.tif]

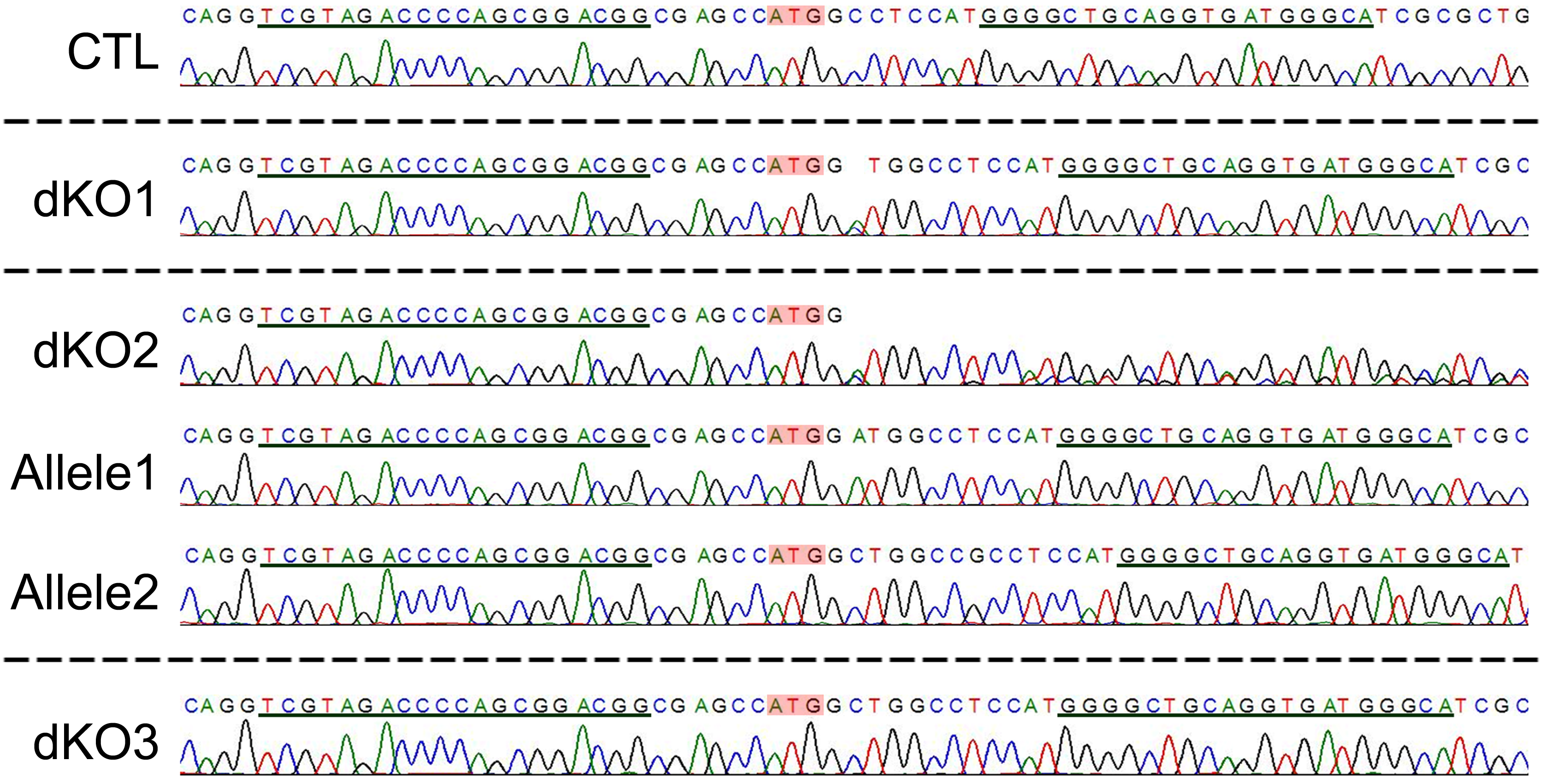

Supplement: S2 Fig — PCR products of the TALEN targeting site from wild-type cells (CTL) and claudin-2 and claudin-4 double knockout clones (dKO1–3) were directly subjected to DNA sequencing analysis. Chromatograms of the sequences of the TALEN targeting site showed a single peak array in the dKO3 clone and mixed peak arrays in the dKO1 and dKO2 clones. PCR products from the dKO2 clone were cloned into a plasmid vector and subjected to sequencing analysis. (TIF) [file pone.0182521.s002.TIF]

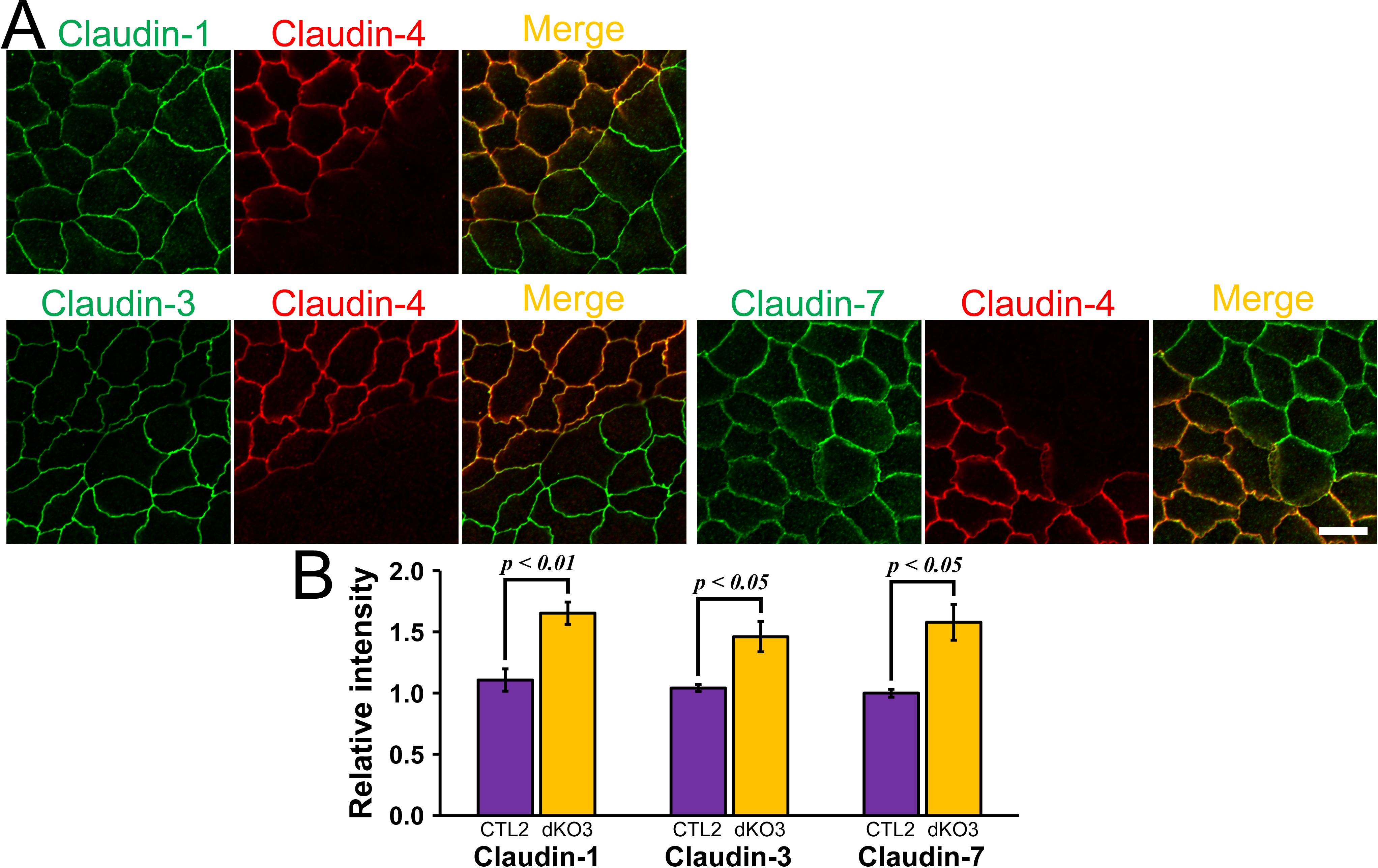

Supplement: S3 Fig — (A) Immunofluorescence analysis of claudins in the co-culture of the claudin-2 knockout clone 2 [13] and the dKO3 clone (claudin-2 and claudin-4 double knockout clone). Scale bar = 10 μm. (B) Quantification analysis of the signal intensity of claudins at TJs in the claudin-2 knockout clone 2 (CTL2) and the dKO3 clone. N = 4 for each experiment. (TIF) [file pone.0182521.s003.TIF]

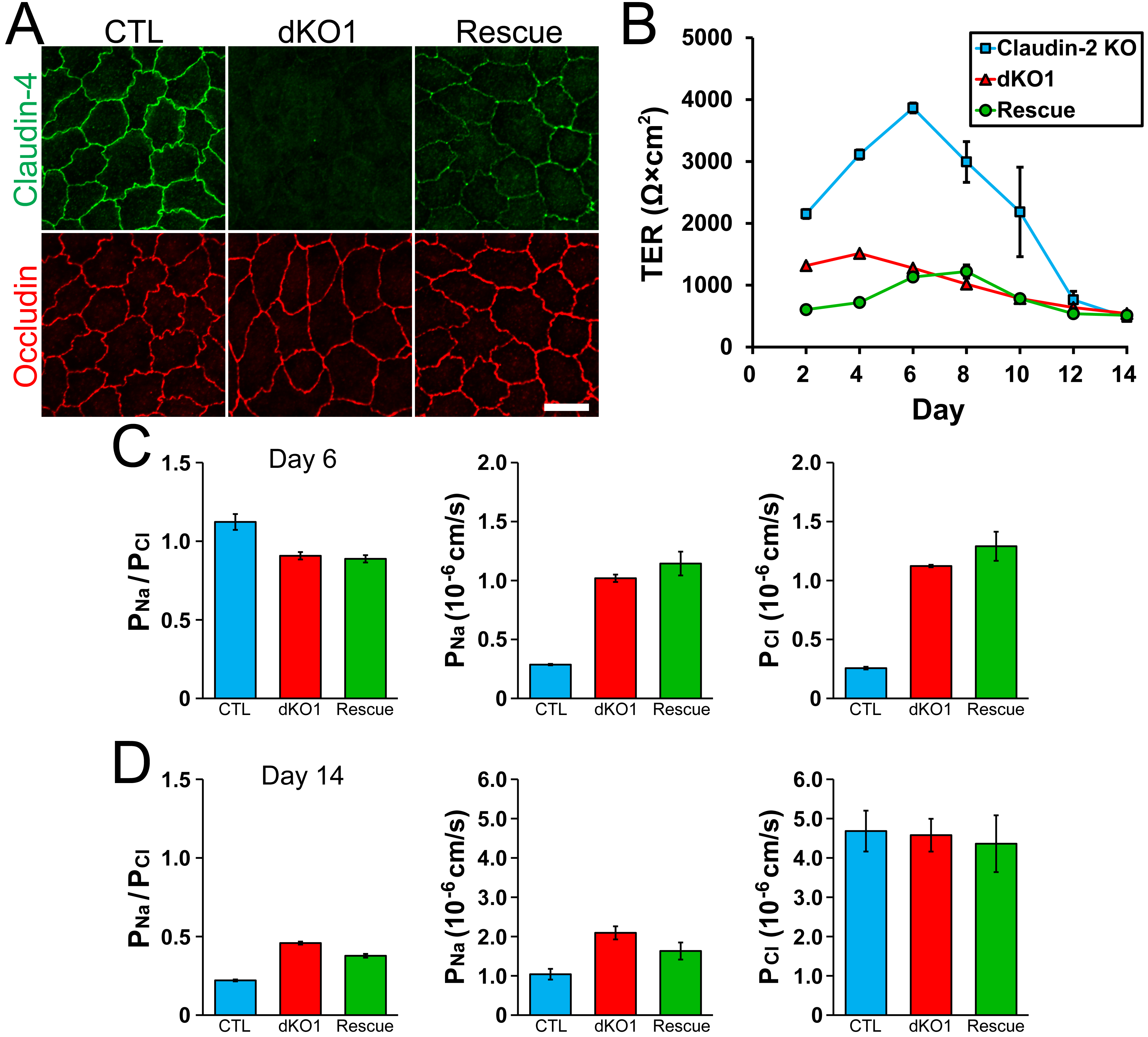

Supplement: S4 Fig — (A) Immunofluorescence analysis of claudin-4 and occludin in claudin-2 knockout clone 1 (CTL), dKO1 clone, and rescue clone. Claudin-4 cDNA was transfected into dKO1 clone, and the clone expressing N-terminally FLAG tagged claudin-4 was established. Scale bar = 10 μm. (B) Time course of TER in claudin-2 knockout clone 1, dKO1 clone, and rescue clone. (C and D) PNa/PCl, PNa and PCl at 6 days (C) and 14 days (D) after the seeding on filter inserts in claudin-2 knockout clone 1, dKO1 clone, and rescue clone. N = 3–4 for each experiment. (TIF) [file pone.0182521.s004.tif]

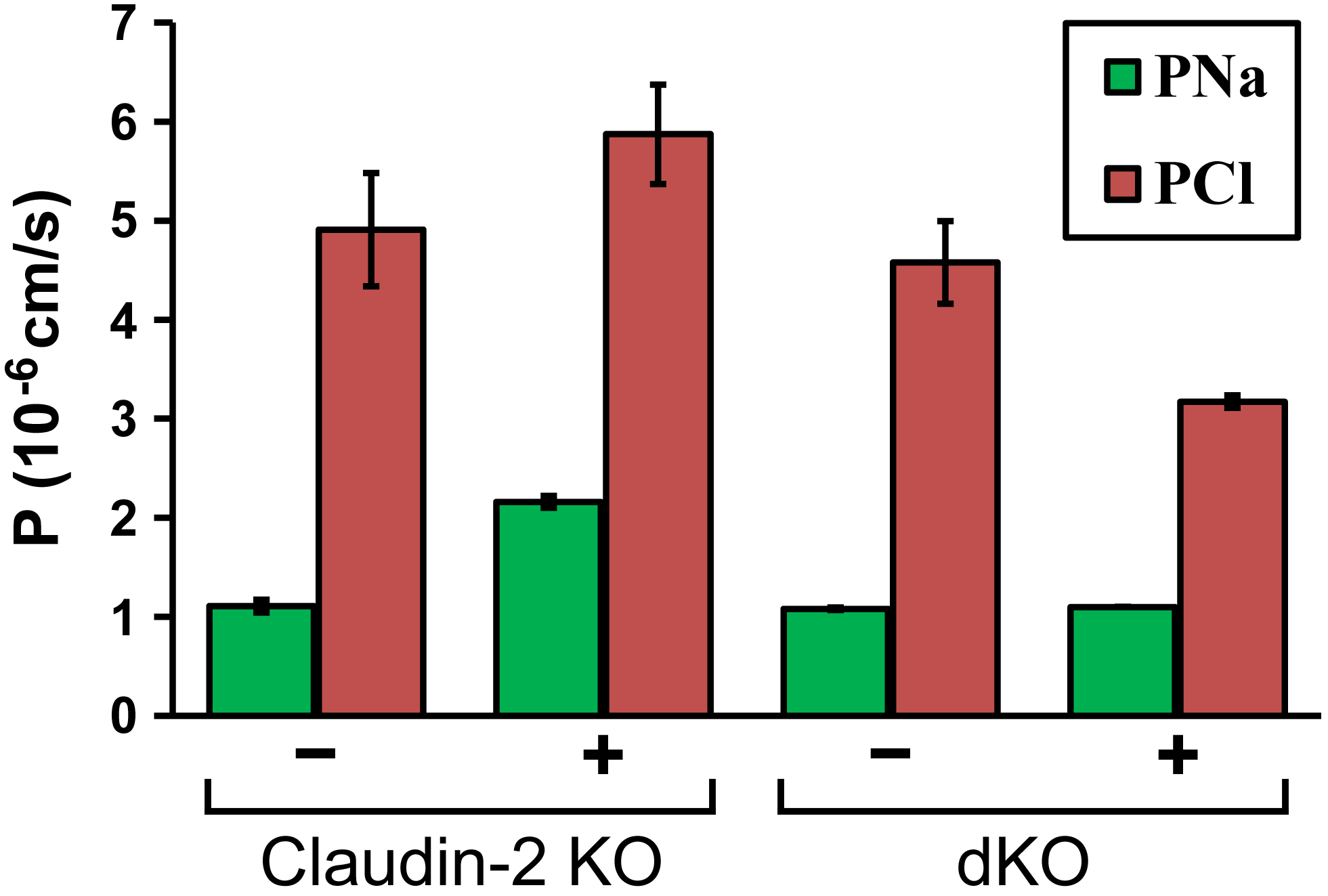

Supplement: S5 Fig — Claudin-2 knockout clone and claudin-2 and claudin-4 double knockout clone were cultured for 14 days on filter inserts. PNa and PCl were measured before (−) and 10 min after (+) the administration of 100μM NPPB and 100μM bumetanide in both the apical and basal sides. (TIF) [file pone.0182521.s005.TIF]

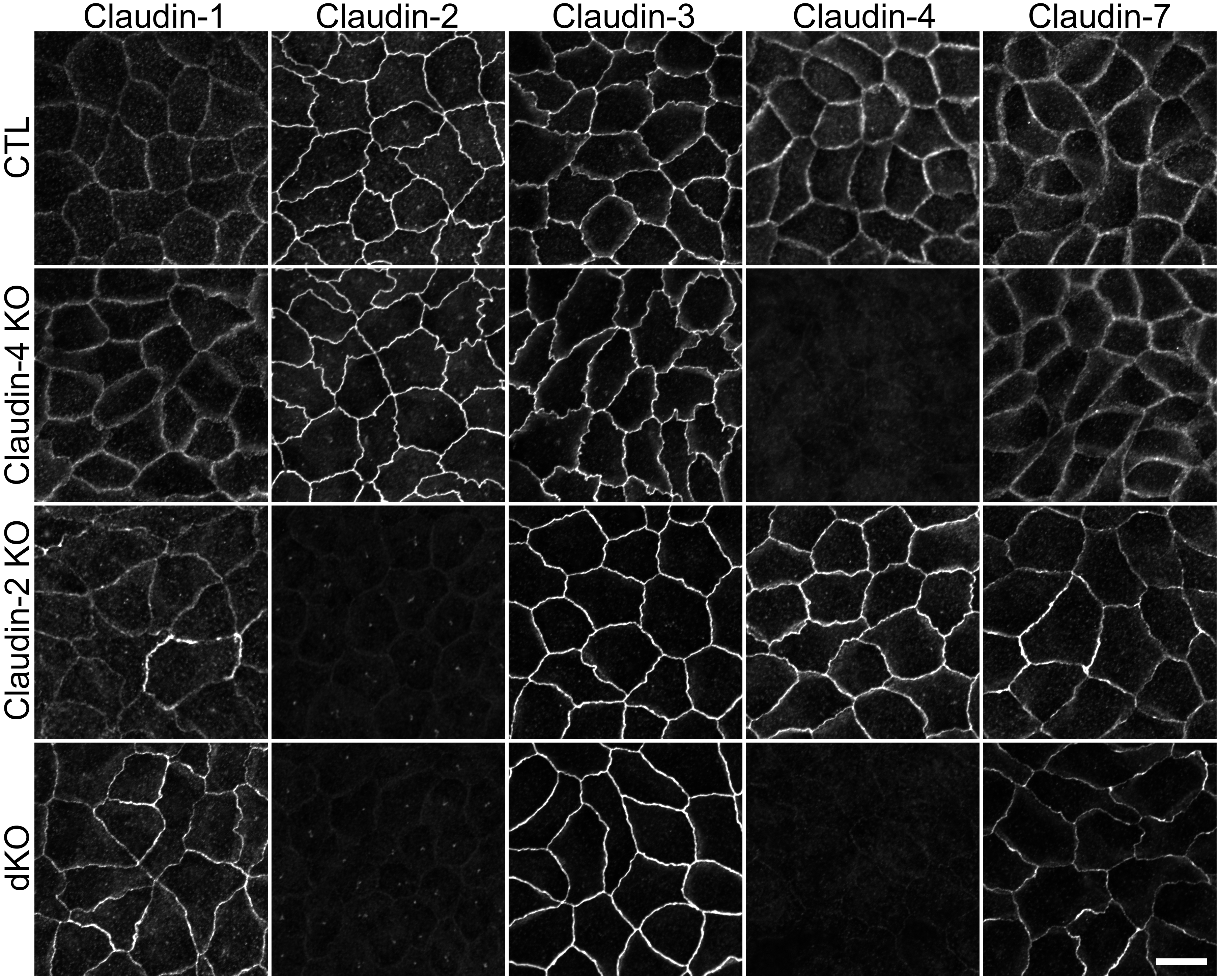

Supplement: S6 Fig — Wild-type MDCK II cells, claudin-4 knockout clone, claudin-2 knockout clone, and claudin-2 and claudin-4 double knockout clone were cultured for 14 days on filter inserts and analyzed by immunofluorescence microscopy for claudins. Scale bar = 10 μm. (TIF) [file pone.0182521.s006.tif]

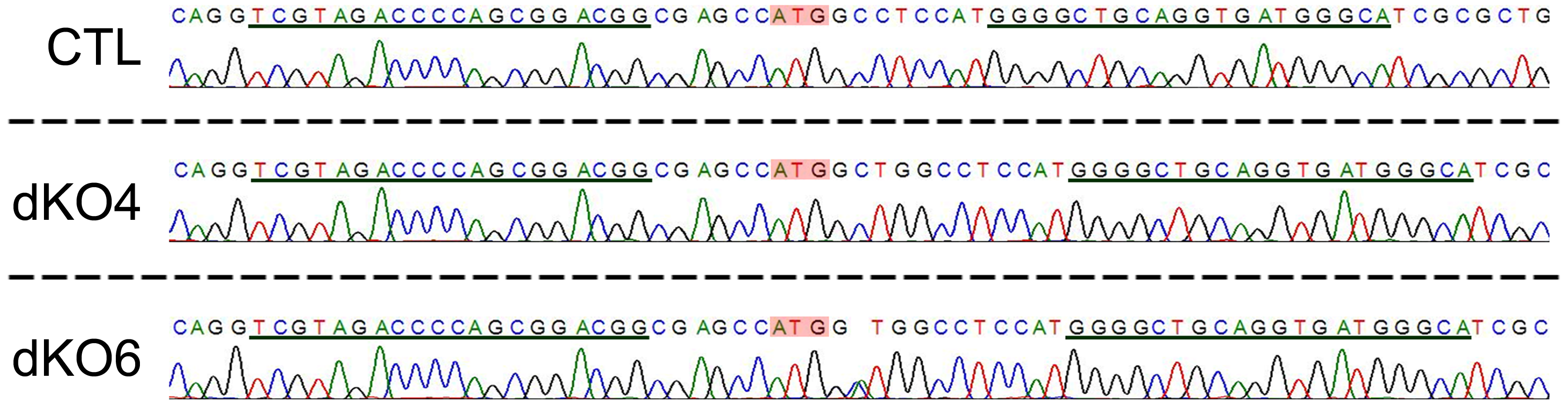

Supplement: S7 Fig — PCR products of the TALEN targeting site from wild-type cells (CTL) and claudin-4 knockout clones (dKO4, dKO6) were directly subjected to DNA sequencing analysis. (TIF) [file pone.0182521.s007.TIF]
